# Ultrabroadband 3D invisibility with fast-light cloaks

**DOI:** 10.1038/s41467-019-12813-2

**Published:** 2019-10-24

**Authors:** K. L. Tsakmakidis, O. Reshef, E. Almpanis, G. P. Zouros, E. Mohammadi, D. Saadat, F. Sohrabi, N. Fahimi-Kashani, D. Etezadi, R. W. Boyd, H. Altug

**Affiliations:** 1 Solid State Physics Section, Department of Physics, National and Kapodistrian University of Athens, Panepistimioupolis, GR-157 84 Athens, Greece; 2 Department of Physics and School of Engineering and Computer Science, University of Ottawa, Ottawa, Ontario K1N 6N5 Canada; 3 Institute of Nanoscience and Nanotechnology, NCSR “Demokritos,” Patriarchou Gregoriou and Neapoleos St., Agia Paraskevi, GR-153 10 Athens, Greece; 4 School of Electrical and Computer Engineering, National Technical University of Athens, Athens, 15773 Greece; 5 Bioengineering Department, EPFL – École Polytechnique Fédérale de Lausanne, 1015 Lausanne, Switzerland; 6 Department of Mechanical Engineering, University of Massachusetts Lowell, 1 University Avenue, Lowell, MA 01854 USA

**Keywords:** Metamaterials, Transformation optics

## Abstract

An invisibility cloak should completely hide an object from an observer, ideally across the visible spectrum and for all angles of incidence and polarizations of light, in three dimensions. However, until now, all such devices have been limited to either small bandwidths or have disregarded the phase of the impinging wave or worked only along specific directions. Here, we show that these seemingly fundamental restrictions can be lifted by using cloaks made of fast-light media, termed tachyonic cloaks, where the wave group velocity is larger than the speed of light in vacuum. On the basis of exact analytic calculations and full-wave causal simulations, we demonstrate three-dimensional cloaking that cannot be detected even interferometrically across the entire visible regime. Our results open the road for ultrabroadband invisibility of large objects, with direct implications for stealth and information technology, non-disturbing sensors, near-field scanning optical microscopy imaging, and superluminal propagation.

## Abstract (web summary)

Three-dimensional invisibility cloaks are either limited in bandwidth or disregard the phase of the impinging wave or work only in specific directions. Here, the authors report that these restrictions can be lifted by using cloaks made of fast-light media where the wave group velocity is larger than the speed of light in vacuum.

## Introduction

The interaction of light and electromagnetic waves with polarized or charged objects leads to the re-radiation and scattering of the wave energy from the objects, making them inherently detectable in the near and/or far field. The ability to suppress this scattering and conceal an object completely from detection has long been the subject of research efforts but it has only been in the past decade, with continued advances in nanotechnology and the field of metamaterials^1–4^, that a wider systematic effort has been inaugurated^5–8^. A variety of invisibility cloaking devices have thus been investigated, including Euclidian^6,7^ and non-Euclidian^8^ transformation-optics cloaks, carpet cloaks^9–16^, and plasmonic^17–19^ and mantle^20,21^ cloaks. However, they all bear inherent limitations that have so far prevented their deployment for broadband, across-the-visible 3D invisibility that preserves the phase of the incident wave—that is, true invisibility.

In particular, Euclidean transformation-optics cloaks provide three-dimensional cloaking for all polarizations but are prohibitively narrowband in the visible regime, with typical bandwidths being on the order of 0.00005% of the visible spectrum^22–24^, and require sophisticated engineering of anisotropic material parameters—both, electric and magnetic, with the latter being challenging to attain in the visible regime. Furthermore, an object wrapped with such cloaks is blind to the surrounding environment, as the incident field does not penetrate in the region of the object—i.e., the object cannot ‘see’ the exterior space through the cloak. Non-Euclidean transformation-optics devices can alleviate bandwidth restrictions but they, too, involve intricate distributions of material parameters in 3D, and do not preserve the phase of the incident wave—rendering the object, in principle, detectable using time-of-flight or interferometric techniques^24^. Two-dimensional quasi-conformal transformation-optics cloaks, known as carpet or ground plane cloaks, exhibit broadband operation but they are inherently two-dimensional leaving an object detectable by measurements in the third dimension, and suffer from the lateral-shift effect^25^. When isolated or placed back-to-back, such cloaks, although broadband, work for only a given polarization of light or at specific directions and/or do not preserve the phase of the incident wave^24^. Plasmonic cloaking, on the other hand, leads to scattering suppression by compensating for the polarization of an object with the opposite polarizability of a shell. It is simpler to build but relies on the use of lossy (particularly in the visible) negative-permittivity materials and is only somewhat more broadband than the transformation-optics (TO) approach^23,24^. Finally, mantle cloaks manage to strongly suppress the scattering from an object using thin frequency-selective surfaces with induced currents that cancel the re-radiated (scattered) wave energy, but owing to their increased complexity they are more suitable in the microwave regime and have bandwidths slightly larger than plasmonic cloaks. Further interesting approaches to scattering cancelation, with similar limitations as the above methods, such as, e.g., the Kerker effect^26,27^ which leads to suppressed backscattering but not to invisibility from all angles, are reviewed in ref. ^24^.

**Fig. 1:**
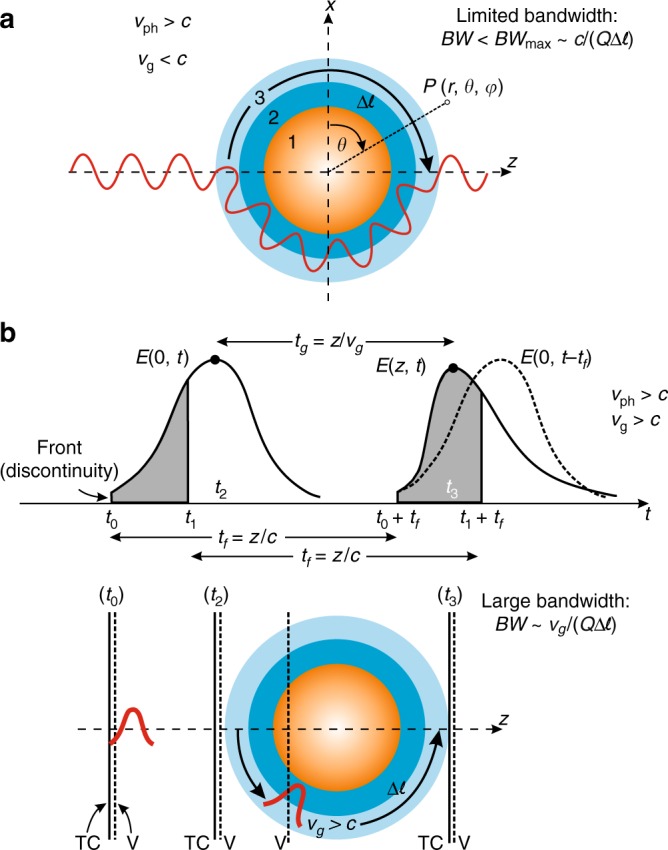
Bandwidth characteristics of conventional and tachyonic invisibility cloaks. **a** In conventional cloaks, be them Euclidean, plasmonic or mantle cloaks, a wavelet propagates with a superluminal phase velocity, *v*_ph_, but subluminal group velocity, *v*_g_, smoothly around a three-dimensional (here, spherical) object, preserving the phase at the exit/right-hand side of the cloak compared with a wavelet propagating in vacuum. This renders the object invisible to the incident radiation but, because *v*_g_ < *c*, over only a limited bandwidth, BW < BW_max_, with BW_max_ being proportional to the vacuum light speed, *c*, and inversely proportional to the extra path, Δ*ℓ*, taken by the wavelet around the object^22^. At visible frequencies, BW_max_ can be extremely narrow, of the order of ~0.00005% of the visible spectrum^22–24^—i.e., the object becomes invisible at, approximately, a single wavelength only. **b** In a tachyonic cloak (TC), both, the phase and the group velocities are superluminal over broad spectral ranges. Here, the peak of (the envelope of) an ultrashort pulse travels faster than the speed of light in vacuum, *c*, in a such a way that a wavefront of the pulse (shown with solid or dashed vertical lines in the lower part) is, at all times, at the same position as the wavefront of a pulse traveling in vacuum (V), despite the fact that the pulse traversing the cloaked object takes a longer route (around the object) Δ*ℓ* compared with a pulse traveling straightly in vacuum. This extra pathlength is, here, balanced out by the correspondingly larger group velocity of the pulse in the cloak (*v*_g_ > *c*), so that the ratio *v*_g_/Δ*ℓ* is maintained large—and, in principle, with no upper bound. The attained bandwidths can, thus, now be extremely broadband, even for phase-preserving cloaking of highly scattering objects in air. There is no violation of relativistic causality with such a scheme because, as explained in the main text and the Supplementary Note 2, only the shaded parts of the input and output pulses, shown in the upper part, are causally connected

From the above considerations it is clear that true invisibility, that is a three-dimensional cloak rendering an object invisible from all angles across the entire visible regime and for all polarizations of light while fully preserving the incident-wave phase and amplitude, has not been attained yet—and, in fact, there are a number of works suggesting that such a feat is probably fundamentally unattainable^22–24,28–30^. Figure 1a conceptually illustrates the often-mentioned reason for why this might be the case: With the cloak wrapped around an object, an incident pulse of duration *τ* = 1/BW, BW being the bandwidth of the pulse, must transverse the extra path Δ*ℓ* around the object in roughly the same time Δ*t* as that needed to propagate straight ‘through’ the object so that the cloak can fully restore the incident field distribution all around the object in, both, amplitude and phase. Since Δ*t* = Δ*ℓ*/*v*_g_, with *v*_g_ being the group velocity of the pulse, and *Q* = *τ*/Δ*t*, where *Q* is a quality factor associated with pulse distortion (usually *Q* ≈ 6 for acceptable levels of distortion)^22^, it immediately follows that BW = *v*_g_/(*Q*·Δ*ℓ*). In the pertinent literature, it is invariably at this point argued that the wave group velocity *v*_g_ must be smaller than the speed of light in vacuum, *c* (refs. ^6–8,22–24,28–30^) from where a maximum bandwidth BW_max_ for invisibility cloaking is ascertained BW < BW_max_ = *c*/(*Q*·Δ*ℓ*) (Fig. 1a). In the visible regime and for macroscopic objects (nanoscopic objects would require a TO cloak with sub-nm engineering of material parameters, which is presently impractical or impossible) this expression directly indicates extremely small cloaking bandwidths, on the order of ~0.00005% of the visible spectrum^22^—with similar order-of-magnitude results obtained on the basis of more detailed analyses^23^.

Here, we show that these seemingly fundamental limitations can be overcome by designing cloaks made of abnormally dispersive media, i.e., media in which the wave group velocity *v*_g_ can become superluminal (*v*_g_ > *c*), infinite or negative (*v*_g_ < 0) over broad bandwidths and without appreciable modification of the superluminal pulse’s shape^31–34^ (Fig. 1b)—i.e., without dispersion destroying the shape of the incident pulse, so that the group velocity fully retains its meaning. Such media have been studied long ago by Sommerfeld^35^ and Brillouin^36^, and more recently in the field of ‘fast light’, and fully comply with relativistic causality and Kramers–Kronig relations^31,32^. In addition, they can indeed lead to breaking scattering causality^37^, where the presence of a scatterer (object) causes the wave to reach the detector faster than in the scatterer’s absence. Cloaks made of these media will be called tachyonic^38^ because they give rise to superluminal light group velocity.

## Results

### Physical insights and rationale

Consider the configuration shown in Fig. 1b, where a dielectric spherical scatterer of radius *r*_1_ and permittivity *ε*_1_—the archetypal object in most 3D invisibility cloaking studies—is cloaked from an incident electromagnetic field (**E**_0_, **H**_0_) by two shells of optical materials with permittivities *ε*_2_, *ε*_3_(*ω*) and thicknesses *r*_2_ – *r*_1_ and *r*_3_ – *r*_2_, respectively. We set *ε*_4_ = *ε* to be the permittivity of the background medium, and we assume that the outer shell (3) is made of a fast-light medium, i.e., a medium supporting superluminal wave group velocities (see Fig. 1b and discussion later on). To obtain a clear physical insight of the requirements for invisibility in the problem at hand, let us initially assume that **H**_0_ (but not **E**_0_) varies slowly in time (∂**H**_0_/∂*t* → 0) (ref. ^39^), with our analysis of the investigated geometries being extended into the full electrodynamic regime later-on based, respectively, on a recursive transfer-matrix formulation and an exact electromagnetic scattering analysis (see Supplementary Notes 3, 4) as well as on the basis of corroborating full-wave finite-element (COMSOL) and integral-equation (CST) methods.

Owing to the azimuthal symmetry of the problem, the electric potential in the four regions, *ϕ*_*e*,*i*_ (*i* = 1–4), will be of the form $\varphi _{e,i}(r,{\it{\vartheta }}) = \mathop {\sum}\nolimits_{n = 0}^\infty {(A_{i,n}r^n + B_{i,n}r^{ - (n + 1)})P_n(\cos {\it{\vartheta }})} ,$ and should obey the continuity relations at *r*_*j*_ (*j* = 1–3): $\varphi _{e,j}(r_j){\it{ }} = \varphi _{e,j + 1}(r_j)$ and $\varepsilon _j(\partial \varphi _{e,j}/\partial r)|_{r = r_j} = \varepsilon _{j + 1}(\partial \varphi _{e,j + 1}/\partial r)|_{r = r_j}.$ From the continuity equations, together with considerations for the well behavior of *φ*_*e*_ at *r* → 0 and *r* → ∞, we arrive at a system of eight equations for determining the eight unknowns of the problem (the coefficients *A*_*i*,1_, *B*_*i*,1_, *i* = 1–4), since it turns out that all other coefficients *A*_*i,n*_, *B*_*i,n*_ with *n* ≠ 1 vanish; see Supplementary Information, SI.

The solution to the above 8 × 8 system leads to the following exact (to all multipole orders) expression for the spatial profile of the field in the region outside of the cloaked object (*r* ≥ *r*_3_):

1 $${\mathbf{E}}_4 = \left( {\frac{{2B_{4,1}}}{{r^3}} - E_0} \right)\,\cos {\it{\vartheta }}{\hat{\bf{r}}}_0 + \left( {E_0 + \frac{{B_{4,1}}}{{r^3}}} \right)\,\sin {\it{\vartheta }}{\hat{\bf{\theta }}}_0.$$

From the above expression, it becomes immediately clear that if we manage to obtain *B*_4,1_ = 0 over a broad spectral range, the electromagnetic field in the region outside the external cloak will become $({\mathbf{E}}_4,{\mathbf{H}}_4) = [E_0( - {\mathrm{cos}}{\it{\vartheta }}{\hat{\mathbf{r}}}_0 + {\mathrm{sin}}{\it{\vartheta }}{\hat{\mathbf{\theta }}}_0),\,{\mathbf{H}}_0] = ( - E_0{\bf{z}}_0,\,{\mathbf{H}}_0) = ({\mathbf{E}}_0,{\mathbf{H}}_0),$ i.e., the incident field will remain completely unperturbed by the cloaked object—a similar situation with the TO cloaks; hence the object becomes invisible to the external radiation, for all 3D angles of incidence, while fully preserving the phase of the incident wave. From the calculations outlined in the Supplementary Note 1, we find that the coefficient *B*_4,1_ is given by:

2 $$B_{4,1} = - {\mathbf{E}}_0r_3^3\frac{{r_2^3(\varepsilon _4 + 2\varepsilon _3)(r_2^3\tau _1 + r_1^3\tau _2) + r_3^3(\varepsilon _3 - \varepsilon _4)(r_2^3\tau _4 - 2r_1^3\tau _3)}}{{2r_2^3(\varepsilon _3 - \varepsilon _4)(r_2^3\tau _1 + r_1^3\tau _2) + r_3^3(\varepsilon _3 + 2\varepsilon _4)(r_2^3\tau _4 - 2r_1^3\tau _3)}},$$

where *τ*_1_ = (*ε*_2_ − *ε*_3_)(2*ε*_2_ + *ε*_1_), *τ*_2_ = (*ε*_1_ − *ε*_2_)(2*ε*_2_ + *ε*_3_), *τ*_3_ = (*ε*_2_ − *ε*_1_)(*ε*_2_ − *ε*_3_), and *τ*_4_ = (2*ε*_2_ + *ε*_1_)(2*ε*_3_ + *ε*_2_). Thus, we find that the coefficient *B*_4,1_ can indeed vanish, as long as the following relation is satisfied:

3 $$r_{2,3}^3 = \frac{{r_2^3}}{{r_3^3}} = \frac{{(\varepsilon _4 - \varepsilon _3)(r_{2,1}^3\tau _4 - 2\tau _3)}}{{(\varepsilon _4 + 2\varepsilon _3)(r_{2,1}^3\tau _1 + \tau _2)}},$$

with 0 < *r*_2,3_ < 1 and $$r_{2,1}^3 = r_2^3/r_1^3\ > \ 1.$$

To allow the incident plane wave propagating through the cloak travel longitudinally with the same effective velocity as that of a plane wave propagating in the absence of the cloak and the object (vacuum), so that there is no waveform distortion and true invisibility can be attained, we may design our cloak such that its outer layer (3) is made of a dispersive fast-light medium, i.e., a medium supporting a superluminal wave group velocity over a broadband—equivalently, making the coefficient *B*_4,1_ zero over broad spectral ranges (see discussion below). In such a fast-light medium the wave propagates with an average (over the pulse’s spectrum) group velocity 〈*v*_g_〉 > *c*. Therefore, although it effectively takes a longer route (around the spherical object) to reach the other side compared to a plane wave propagating straight through vacuum, it can still reach the side behind the cloaked object (e.g., time-point *t*_3_ in the lower part of Fig. 1b) simultaneously with a plane wave propagating in vacuum, so that no shadow or waveform distortion arises. Importantly, in sharp contrast to current 3D, phase-preserving cloaking designs where only the phase velocity at a particular frequency is superluminal^6,7,17,20^, making those designs inherently narrowband, here the presence of superluminal wave group velocities implies much increased bandwidth^31,32,34^ and is the key to attaining broadband, three-dimensional, phase-preserving invisibility.

In this tachyonic cloak, the condition 〈*v*_g_〉 > *c* does not violate relativistic causality not because (as it is sometimes argued) the group velocity loses its meaning in the presence of dispersion and absorption, but because—as shown in the SI and explained pictorially in Fig. 1b—the signal-front velocity (the velocity with which the ‘turn on’ discontinuity in the incident pulse propagates) is exactly equal to *c*. In other words, the signal velocity, which contains the sudden (‘surprising’) turn-on information (discontinuity) is not superluminal, despite 〈*v*_g_〉 > *c*, in accordance with causality. Physically, as shown in Fig. 1b, because 〈*v*_g_〉 > *c*, the output pulse traveling through the cloak is reshaped, with its peak being shifted to earlier times compared to a pulse propagating in vacuum. We note from Fig. 1b (see also SI) that only the shaded parts of the incident and output pulses are causally connected, i.e., only the shaded part of the incident pulse in the interval [*t*_0_, *t*_1_] determines causally the shaded part of the exit pulse in the interval [*t*_0_ + *z*/*c*, *t*_1_ + *z*/*c*]. Since the shaded part of the incident pulse in the interval [*t*_0_, *t*_1_] does not include the peak of the incident pulse, but the shaded part of the output pulse in the interval [*t*_0_ + *z*/*c*, *t*_1_ + *z*/*c*] does include the peak of the output pulse, the two peaks are not causally connected—and, therefore, the time delay between them, *t*_g_ = *z*/*v*_g_, can indeed by superluminal, *t*_g_ < *t*_f_ *=* *z*/*c*. The above observations, thus, completely explain—physically, but also on the basis of the rigorous calculations shown in SI (sec. 2)—how superluminal wave group velocities over broad bands are obtained in the outer shell of this tachyonic cloak, without violation of relativistic causality.

### Design considerations

A practical demonstration of our scheme utilizes fast-light media, which have in the recent past been extensively investigated and demonstrated experimentally^31–34^, and which can be extremely broadband^31,32,41^, facilitating distortionless pulse propagation with negligible amplitude change^31,32,34^. For instance, far from resonance in an inverted (amplifying) medium [(*ω*_12_ − *ω*)^2^ ≫ *γ*^2^], the real part of the refractive index is^31^ *n*_R_(*ω*) = *n*_i/v_ − (*Nwe*^2^*f*)/[(4*mε*_0_*ω*_12_)(*ω*_12_ − *ω*)], where *n*_i/v_ < 1 is the background refractive index owing to interband transitions or valence electrons, *N* is the number density of atoms, *w* = (*N*_2_ − *N*_1_)/*N* > 0, with *N*_1_, *N*_2_ being respectively the populations of the lower and upper energy levels, *e*, *m* being respectively the electron charge and mass, *f* the oscillator strength, and *ω*_12_ the transition frequency between the lower, |1〉, and upper, |2〉, energy levels. At frequencies sufficiently far from *ω*_12_, the group velocity in such an inverted medium, *v*_g_ = *c*/(*n*_R_ + *ω*d*n*_R_/d*ω*), is thus superluminal (*v*_g_ > *c*) over broad spectral ranges and with negligible dispersion. One may also deploy a gain doublet configuration^34^, where in the broad spectral region between two gain peaks there is strong anomalous dispersion but negligible gain (or absorption). In such a broad spectral region, the group velocity *v*_g_ can significantly exceed *c*, and can even become negative^33,34,40,41^, with insignificant change in either the amplitude or the shape of the pulse.

Furthermore, any other method allowing broadband superluminal light propagation—the key ingredient of our present tachyonic cloaking scheme—is also sufficient for our purposes. One such, making use of semiconductor optical amplifiers (SOAs), has been detailed in a series of pertinent recent works—both, theoretically and experimentally^42–45^. Remarkably, it allows for attaining fast (superluminal) light over very broad bandwidths, at optical wavelengths, resulting in time advances of over several pulse-widths compared with the arrival time of light propagating ordinarily (*c*/*n*) in the same media. This scheme is also readily extendable to visible wavelengths, e.g., using standard InGaN/GaN quantum-well based devices^46,47^, which have nowadays been established as the fundamental component for solid-state lighting. A further method for attaining ultrabroadband superluminal group velocities exploits, so-called non-Foster, epsilon-near-zero metamaterials, where the dispersion of ordinary passive metamaterials can be compensated for with the ‘inverse’ dispersion of non-Foster elements, resulting in ultrabroadband behavior^48,49^—even in the optical regime^49,50^. These principles have successfully been exploited in the design of optical metamaterials where deep-subwavelength inclusions controllably tailor the dispersive properties of an established metamaterial structure, thereby producing an ultrabroadband low-loss optical device with any desired response—including Re{*ε*_eff_} < 1 and Re{*μ*_eff_} < 1 (superluminality)^50^. This is, therefore, a powerful and versatile strategy for realizing a wide range of broadband optical devices that exploit the unique properties of epsilon-near-zero metamaterials. Exploiting such media, one may achieve broadband tachyonic cloaking over any desired frequency band, so long as the superluminality condition (average, dispersive, group velocity larger than the speed of light in vacuum, 〈 *v*_g_〉 > *c*) is attained over the desired bandwidth, together with the recursive optimization (optimized material parameters) methodology detailed in the Supplementary Information (notes 3, 4).

### Simulation results

**Fig. 2:**
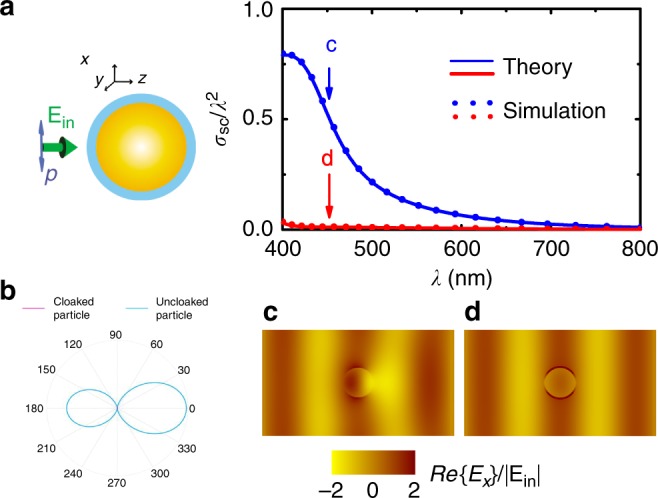
Ultrabroadband tachyonic cloaking. **a** Normalized scattering cross section of the uncloaked (blue line) and cloaked (red line) spherical object shown in the left inset graphic. The object has relative permittivity *ε*_r_ = 5 and magnetic permeability *μ*_r_ = 1. The thickness of the cloak is only 10 nm, and inside it the average (dispersive) group velocity is ten times larger than the speed of light in vacuum (〈*v*_g_〉~10*c*). Solid lines correspond to analytical calculations (see Supplementary Notes 3, 4), while the symbols correspond to finite-element (Comsol) and integral-equation (CST) simulations that precisely coincide. **b** Polar plot of the scattering from the uncloaked (blue line) and cloaked (red dot, at the center) spherical object at an incident wavelength *λ* = 450 nm. **c**, **d** Electric-field profile (*E*_*x*_ component) in the *xz* plane, including the center of the spherical object, for the uncloaked (**c**) and for the cloaked (**d**) particle, respectively, at an incident wavelength *λ* = 450 nm. Note in (**c**) the perturbed field exiting the object

To clearly illustrate the effectiveness of this approach, we use the analytic methodologies outlined above and in the SI to design such a tachyonic cloak for various 3D geometries, which we then simulate using full-wave causal numerical computations (both, finite-element and integral-equation based). The first structure (Fig. 2) is made of a strongly scattering (in the visible regime) dielectric sphere, of relative permittivity *ε*_r_ = 5, surrounded by a 10-nm-thick far-off-resonance inverted (gain) medium with a refractive index given by the expression for *n*_R_ above, inside which the averaged (over the visible band) group velocity is 〈*v*_g_〉 ~10*c* (superluminal/fast-light propagation). The whole configuration is embedded in air. Figure 2a, b shows that the uncloaked sphere indeed scatters strongly, particularly at the angles 0° (in front of the object) and 180° (behind the object), following a well-known double-lobe scattering pattern. Insertion of the tachyonic double-layer cloak around the object leads to complete suppression of scattering from all angles, i.e., to invisibility, reducing the scattering cross section of the object to virtually zero. We discern from Fig. 2d that the field outside the cloaking region remains completely unperturbed, in accordance with Eqs. (1), (3) and the associated discussions therein and in the SI (secs. 3 and 4), rendering the object undetectable in, both, the far- and the near-field.

**Fig. 3:**
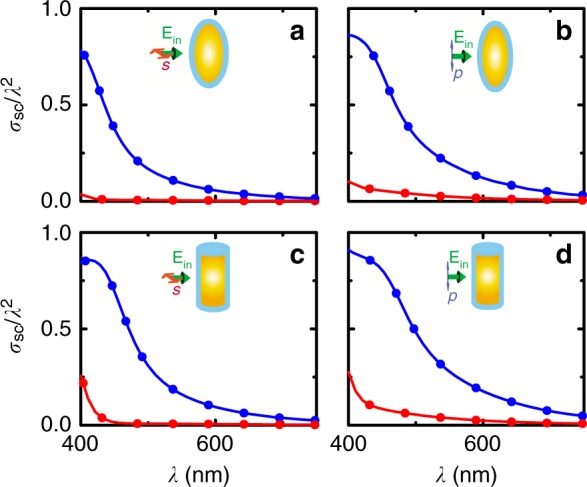
Omni-polarization tachyonic cloaking of non-spherical 3D objects. Here, too, as in Fig. 2, the thickness of the cloak is, in all cases, only 10 nm, and inside it the average (dispersive) group velocity is ten times larger than the speed of light in vacuum (〈*v*_g_〉 ~10*c*)—i.e., the tachyoni*c* cloaks are similar to the one used for the spherical object in Fig. 2, without any additional customization or optimization. **a** Normalized scattering cross section of an uncloaked (blue line) and cloaked (red line) spheroid object, under *s* polarization of light as shown in the inset. **b** Normalized scattering cross section of the uncloaked (blue line) and cloaked (red line) spheroid object, under *p* polarization of light as shown in the inset. **c** Normalized scattering cross section of the uncloaked (blue line) and cloaked (red line) three-dimensional cylindrical object (of finite height), under *s* polarization of light as shown in the inset. **d** Normalized scattering cross section of the uncloaked (blue line) and cloaked (red line) cylindrical object, under *p* polarization of light as shown in the inset. Solid lines correspond to finite-element simulations, while the symbols correspond to integral-equation method solutions. Note in all cases the attainment of ultrabroadband, across-the-visible cloaking performance

Most importantly, and as expected from the above discussions, by virtue of the attained superluminal group velocities in the outer shell, this cloaking performance is extremely broadband (Fig. 2a), giving rise to practically flat-zero scattering across the entire visible regime (free-space wavelengths 400–700 nm)—including the hard-to-cloak forward and backward directions, which as shown in Fig. 2b give rise to maximum scattering of the uncloaked object. This requirement is essential for the realization of true, 3D invisibility, and is herein obtained in a fashion that fully preserves the phase of the incident wave (cf. Fig. 2d), so that the object and the tachyonic cloak cannot be detected using interferometric or time-of-flight techniques. Similar results are obtained for other, nontrivial, 3D geometries, too, such as for spheroids (Fig. 3a, b) and finite-height cylinders (Fig. 3c, d), for both polarizations of the incident light, thereby proving the generality of the proposed scheme—i.e., of the fact that with this ‘customized cloaking’ methodology any object, of arbitrary geometry, can be made invisible across the entire visible regime. Furthermore, for mildly superluminal group velocities, e.g., 〈 *v*_g_ 〉 |^outer-shell^ ~ 2.236*c*, we may see from Fig. S4 of the SI that the radius of the cloaked object can, accordingly, be increased by a corresponding factor of ~2 while maintaining the cloak’s ultrabroadband performance, in line with the discussion provided earlier on the attained cloaking bandwidths [BW = *v*_g_/(*Q*·Δ*ℓ*)]. However, it is to be noted that even larger group velocities are realistically attainable, including infinite and negative group velocities^31–34,40,41^, in which case the size of the cloaked object (~Δ*ℓ*) may correspondingly increase further, without compromising the obtained bandwidths. The presented scheme, and its associated (analytical and numerical) methods of analyses, are thus not restricted to small particles: All (TE and TM) resonances are individually resolved, and suppressed (see Suppl. notes 3, 4), and if the size, ‘Δℓ’, of the object increases, the cloak’s bandwidth performance (‘BW’) can still be extremely broadband so long as the group velocity in the cloak’s layers is increased accordingly [since BW ~ *v*_g_/(*Q*·Δ*ℓ*)]. Thus, there are, in principle, no size restrictions as to how large an object can become using this methodology. Furthermore, even arbitrarily- (not just spherically- or cylindrically-) shaped objects can be cloaked over broad bandwidths, as shown in Fig. 3a, b and Supplementary Fig. 5. We would here wish to note that one of the prime objectives of the present work is to solve the key ‘bandwidth issue’ of present-day cloaking devices^22–24^—not to resolve simultaneously the ‘bandwidth’ and ‘size’ issues^28^. The combined latter, and the solution to it on the basis of a suitable adaptation of the present (superluminality based, yet relativistically causal) methodology, will be the subject of a separate work.

### Broadband non-disturbing sensors

**Fig. 4:**
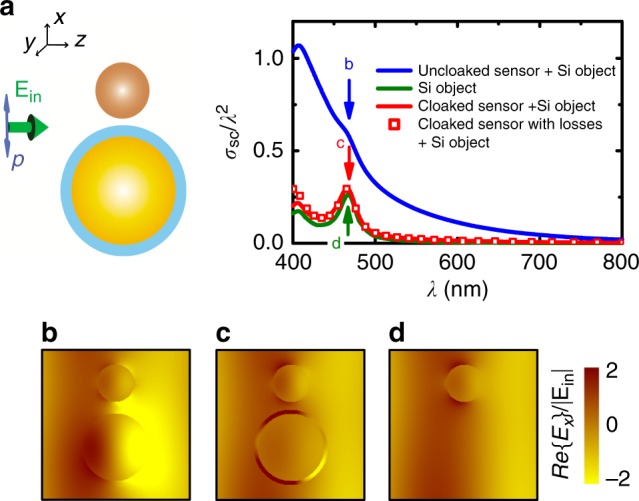
‘See-through’ invisible tachyonic sensor. **a** Plotted vs. visible wavelengths is the total cross section (blue line) of the combined system of an uncloaked detector (lower part of left inset, without the cloak) in close proximity (30 nm distance) to a Si object (top part of inset)—where, both, the dispersive and absorptive properties of Si have been taken into account according to experimental data [e.g., D. E. Aspnes, “Properties of silicon,” *EMIS Datareviews*^59^]. Also shown are the total cross section (*σ*_tot_ = *σ*_sc_ + *σ*_abs_) of the two-object system shown in the inset, illuminated with light polarized parallel to the system’s axis, without (red solid line) and with (red symbols) realistic sensor losses (Im{*ε*_sensor_} = 0.01), for a gap distance of 20 nm, and the total cross section for the isolated Si sphere (green). **b**–**d** Corresponding electric-field profiles for the cases pointed out with arrows in (**a**), calculated on the basis of finite-element (Comsol) full-wave causal simulations. Note in (**c**) the penetration of the incident field inside the region of the cloaked object, unlike transformation-optics cloaks, making the object capable of ‘seeing through’ the cloak its surrounding environment, with virtually no perturbation of the field around the sensed (top) object (compare the field around the object in (**c**) and in (**d**))

The fact that the incident wave penetrates the interior region of the cloak (cf. Fig. 2d)—unlike, e.g., transformation-optics designs^6–8^—suggests that this scheme can also be used for designing minimally invasive sensors^51–53^, over ultrabroadband frequency regimes. Indeed, Fig. 4 clearly shows how a three-dimensional detector, surrounded by a tachyonic cloak that makes the detector invisible, perturbs only faintly the scattering by an external spherical (Si) object—i.e., the scatterings of the combined system ‘cloaked detector + object’, and of the object alone, are almost identical across the entire visible regime, even for the case of a lossy cloaked detector (accounting for the energy lost during the sensing process) as shown with the red symbols in Fig. 4a. Thus, it is possible, using this scheme, to effectively cloak a detector, over ultrabroadband regimes, and still be able to detect an external object since the radiation scattered by the object penetrates the hidden region, making the object detectable. To our knowledge, such a functionality (‘invisible’ sensors across the entire visible band) has not been established before for any invisibility cloaking scheme^51–53^, including scattering-cancellation ones, mainly owing to the rather inherently narrowband responses of those schemes.

## Discussion

Finally, it is interesting to inquire as to what happens when the object is not stationary but instead moves; for instance, how fast should its movement be before it can be detected (i.e., not cloaked)? To this end, we note from Fig. 1b, and from the Supplementary Note 2, that the wave signal corresponding to sharp movements of the object (cf. front discontinuity in Fig. 1b) does not travel superluminally, but at a speed equal to *c*; therefore, according to what has been explained above, infinitely sharp movements cannot be cloaked over broad bandwidths. However, this case is not of much concern because infinitely fast movements of an object do not occur in practice, owing to its inertia^31,41^. For slower movements of the object, we may use Supplementary Equation 17 to show that the electric field at the exit of the tachyonic cloak will, to a good approximation, be given by:

4 $$E(z,t) = E\left( {0,t - \frac{z}{c}} \right) - {\int}_{(z/c)}^t {\frac{{J_1\left( {\omega {\mathrm{p}}\sqrt {t^{\prime 2} - \left( {\frac{z}{c}} \right)^2} } \right)}}{{\sqrt {t\prime ^2 - \left( {\frac{z}{c}} \right)^2} }}\omega _{\mathrm{p}}\left( {\frac{z}{c}} \right)} E(0,t - t^\prime ){\mathrm{d}}t^\prime ,$$

where *J*_1_ is the ordinary Bessel function. Considering that *J*_1_(*t*) → [2/(π*t*)]^1/2^cos(*t* − 3π/4) for *t* ≫ 1, Eq. (4) and Supplementary Note 2 show that while the abrupt discontinuity of the entering pulse exits the cloak only after time *t*_f_ = *z*/*c*, slower movements of the object, occurring at later times in Fig. 1b (e.g., at *t* = *t*_2_ in Fig. 1b), appear at the exit of the cloak at superluminal times (e.g., at *t* = *t*_3_ in Fig. 1b, with *t*_3_ − *t*_2_ = *z*/*v*_g_ < *z*/*c*), and can therefore —according to what has been discussed above—be cloaked over very broad bandwidths. Essentially, for the right choice of parameters in the active (gain) region of the tachyonic cloak(s), the amplitude and time duration of the Brillouin forerunners are drastically minimized—cf. the time-domain simulations snapshots of Figs. 2d and 4c—leading to the attainment of unusually broadband true-cloaking performance.

The tachyonic cloaks introduced and studied here form a recipe for the attainment of ideal invisibility—that is, completely hiding (even interferometrically) an object in air, from all angles in three dimensions and across the entire visible band, while allowing the cloaked object to fully see through the cloak the surrounding environment. It suggests that what was until now considered to be a fundamental limitation of 3D, phase-preserving cloaking devices—their narrow bandwidth performance^22–24,28–30^—may, in fact, be overcome by designing cloaks that support superluminal group velocities. Possible applications include ‘invisible’ sensors that can detect changes in the surrounding environment without interfering with it^51–53^, near-field scanning optical microscopes (NSOMs) with concealed tips for the minimally disturbing imaging of nanoparticles, polymer blends, porous silicon and biological systems^39,51^, electromagnetically camouflaged antennas^4,41^, manipulation of mechanical^54^, optical, sound and heat^55^ waves for information processing, satellite, heat-control and nanotechnology applications^1–4,24,39,56,57^, as well as applications of fast light and superluminal propagation^31,32^, including buffering, regeneration and time-windowing of optical data, ultrahigh-precision spectrometry and interferometry (e.g., for the detection of gravitational waves^58^), rotation sensors based on laser gyros, and laser radars.

## Supplementary information

Supplementary Information

## Data Availability

All relevant dataset generated during and/or analyzed in current study are available from the authors.
