# Supplementary material for: Ultrabroadband 3D invisibility with fast-light cloaks
Source: Nat Commun. 2019 Oct 24;10:4859. doi: 10.1038/s41467-019-12813-2 (PMC6813312; doi:10.1038/s41467-019-12813-2)
Supplement: Supplementary file 1 — Supplementary Information [file 41467_2019_12813_MOESM1_ESM.pdf]

Supplementary Information

Ultrabroadband 3D invisibility with fast-light cloaks

K. L. Tsakmakidis, O. Reshef, E. Almpanis, G. P. Zouros, E. Mohammadi, D. Saadat, F. Sohrabi, N. Fahimi-Kashani, D. Etezadi, R. W. Boyd, and H. Altug

correspondence to: ktsakmakidis@phys.uoa.gr

1. Derivation of Eqs. (1) and (2) of the main text

A lightfield polarized across the x-axis ($\mathbf{E}_0 = -E_0\hat{\mathbf{x}}_0$) is incident from free space on a double-shell tachyonic cloak hiding a spherical object of radius r_1, as shown in Supplementary Fig. 1 below. For certainty, and for the sake of obtaining exact analytic expressions, let us assume that the magnetic field $\mathbf{H_0}$ (but not the electric field $\mathbf{E_0}$) of the incident lightfield varies slowly in time $(\partial \mathbf{H}_0 / \partial t \to 0)$ (Ref. [39]), with the results being fully extendable to faster variations of $\mathbf{H_0}$ provided that the group velocity v_g of the lightwave at the outer shell (3) increases correspondingly – so that the bandwidth $BW = v_g/(Q\cdot\Delta\ell)$ may remain large (see discussions in the main text). Owing to the azimuthal symmetry of the problem, the electric potential in the four regions, $\varphi_{e,i}$ (i = 1–4), will be of the form $\varphi_{e,i}(r,\theta) = \sum_{n=0}^{\infty}(A_{i,n}r^n + B_{i,n}r^{-(n+1)})P_n(\cos\theta)$, and should obey the continuity relations at r_j (j = 1–3): $\varphi_{e,j}(r_j) = \varphi_{e,j+1}(r_j)$ and $\varepsilon_j(\partial\varphi_{e,j}/\partial r)|_{r=r_j} = \varepsilon_{j+1}(\partial\varphi_{e,j+1}/\partial r)|_{r=r_j}$. Furthermore, the following conditions apply on physical grounds:

- The potential $\varphi_{e,1}$ should be finite at $r = 0$, thus: $\varphi_{e,1}(r \le r_1, \theta) = \sum_{n=0}^{\infty} A_{1,n} r^n P_n(\cos\theta)$.
- At large distances ($r \to \infty$) the electric field in region 4 should be equal to $\mathbf{E}_0$. Since, for $r \to \infty$, $\varphi_{e,4}(r,\theta) = \sum_{n=0}^{\infty} A_{4,n} r^n P_n(\cos\theta)$, and for all angles θ it is $\mathbf{E} = -\frac{\partial\varphi}{\partial r}\hat{\mathbf{r}}_0 - \frac{1}{r}\frac{\partial\varphi}{\partial\theta}\hat{\boldsymbol{\theta}}_0$ and $\mathbf{E}_4 = -E_0\cos\theta\hat{\mathbf{r}}_0 + E_0\sin\theta\hat{\boldsymbol{\theta}}_0$, it readily follows that $A_{4,1} = E_0$, and $A_{4,n} = 0$ for all $n \ge 2$. If we further take for reference surface for the potential the surface $\theta = \pi/2$ [$\varphi_4(r, \pi/2) = 0$], we also readily obtain: $A_{4,0} = 0$, and $B_{4,n} = 0$ for all $n \ge 0$ & even.
- From the above consideration for $\varphi_{e,4}$ and the continuity relations at $r = r_3$, we readily

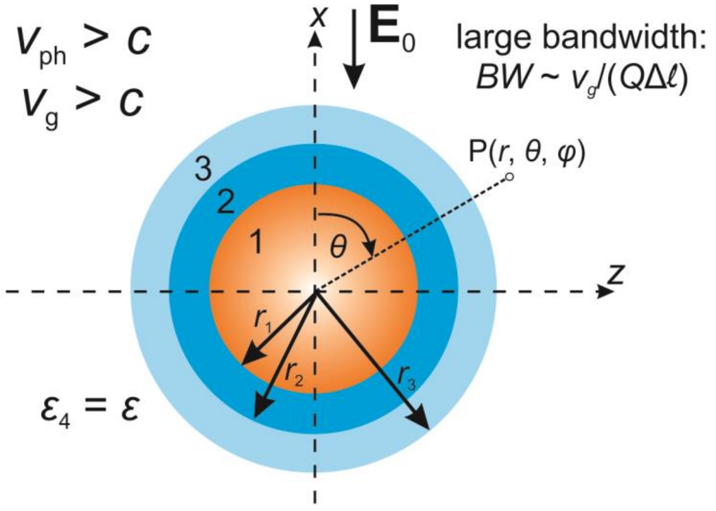

Supplementary Fig. 1. Optogeometric configuration. Double-shell tachyonic cloak (shells 2 and 3) making a spherical object of radius r_1 invisible over a bandwidth $BW \sim v_g/(Q\Delta\ell)$, where the parameters v_g, Q and $\Delta\ell$ are defined in the main text. Shown is also the polarization of the incident lightfield.

obtain: $A_{3,n} = B_{3,n} = B_{4,n} = 0$ for $n \geq 0$ and $n \neq 1$, and: $A_{3,1} + B_{3,1}r_3^{-3} = A_{4,1} + B_{4,1}r_3^{-3}$ (with $A_{4,1} = E_0$, as explained above), and: $\varepsilon_3(A_{3,1} - 2B_{3,1}r_3^{-3}) = \varepsilon(E_0 - 2B_{4,1}r_3^{-3})$.

Likewise, by applying the continuity relations at the other two interfaces ($r = r_1$ and $r = r_2$), we find that all coefficients $A_{i,n}$, $B_{i,n}$ with $n \neq 1$ vanish. Thus, the electric potential in the four regions is simply given by: $\varphi_{e,i} = (A_i r + B_i r^{-2})\cos\theta$ (i = 1–4), and application of the continuity relations leads to the following 8×8 system of equations:

$$\begin{cases} B_1 = 0 \quad (\varphi_{e,1}\ \text{finite at}\ r = 0) & \text{(S1)} \\ A_1 = A_2 + \dfrac{B_2}{r_1^3} & \text{(S2)} \\ A_2 + \dfrac{B_2}{r_2^3} = A_3 + \dfrac{B_3}{r_2^3} & \text{(S3)} \\ A_3 + \dfrac{B_3}{r_3^3} = E_0 + \dfrac{B_4}{r_3^3} & \text{(S4)} \\ A_4 = E_0 \quad (-\nabla \lim_{r\to\infty} \varphi_{e,4} = -E_0 \hat{\mathbf{x}}_0) & \text{(S5)} \\ \varepsilon_1 A_1 = \varepsilon_2 \left(A_2 - \dfrac{2B_2}{r_1^3} \right) & \text{(S6)} \\ \varepsilon_2 \left(A_2 - \dfrac{2B_2}{r_2^3} \right) = \varepsilon_3 \left(A_3 - \dfrac{2B_3}{r_2^3} \right) & \text{(S7)} \\ \varepsilon_3 \left(A_3 - \dfrac{2B_3}{r_3^3} \right) = \varepsilon \left(E_0 - \dfrac{2B_4}{r_3^3} \right) & \text{(S8)} \end{cases}$$

Furthermore, since (as mentioned above) $\mathbf{E} = -\dfrac{\partial \varphi}{\partial r}\hat{\mathbf{r}}_0 - \dfrac{1}{r}\dfrac{\partial \varphi}{\partial \theta}\hat{\boldsymbol{\theta}}_0$ and $\varphi_{e,4} = (A_4 r + B_4 r^{-2})\cos\theta$, the electric field in the region (4) outside the cloaked object will be given by the following exact (to all multipole orders) expression:

$$\mathbf{E}_4 = \left(\frac{2B_4}{r^3} - E_0 \right)\cos\theta \hat{\mathbf{r}}_0 + \left(E_0 + \frac{B_4}{r^3} \right)\sin\theta \hat{\boldsymbol{\theta}}_0, \qquad \text{(S9)}$$

which is Eq. (1) in the main text. The solution to the above system of equations involves tedious calculations, and because of Eq. (S9) (and the associated discussions in the main text) below we provide the final, compact expression for (only) the coefficient B_4:

$$B_4 = -E_0 r_3^3 \frac{r_2^3(\varepsilon_4 + 2\varepsilon_3)(r_2^3\tau_1 + r_1^3\tau_2) + r_3^3(\varepsilon_3 - \varepsilon_4)(r_2^3\tau_4 - 2r_1^3\tau_3)}{2r_2^3(\varepsilon_3 - \varepsilon_4)(r_2^3\tau_1 + r_1^3\tau_2) + r_3^3(\varepsilon_3 + 2\varepsilon_4)(r_2^3\tau_4 - 2r_1^3\tau_3)}, \qquad \text{(S10)}$$

where $\tau_1 = (\varepsilon_2 - \varepsilon_3)(2\varepsilon_2 + \varepsilon_1)$, $\tau_2 = (\varepsilon_1 - \varepsilon_2)(2\varepsilon_2 + \varepsilon_3)$, $\tau_3 = (\varepsilon_2 - \varepsilon_1)(\varepsilon_2 - \varepsilon_3)$, and $\tau_4 = (2\varepsilon_2 + \varepsilon_1)(2\varepsilon_3 + \varepsilon_2)$. Equation (S10) above is used as Eq. (2) in the main text.

2. **Superluminal light propagation in the tachyonic cloak and maintenance of relativistic causality**

To attain the broadband cloaking response shown in Fig. 3 of the main text, a short pulse of bandwidth B must travel superluminally, with a group velocity $v_g > c$, inside the cloak (and around the object[22]) so that there is no appreciable delay compared with a pulse that travels straightly in vacuum and reaches the exit side of the cloak. Both, the amplitude and the phase of the two pulses must (ideally) be exactly the same, so that the object and the cloak cannot be detected using interferometric or time-of-flight measurements. In this section we shall theoretically show that such a feat is, indeed, possible (the corresponding numerical corroboration, obtained from full-wave causal simulations, is presented in Figs. 2 and 3 of the main text) without violation of relativistic causality[31].

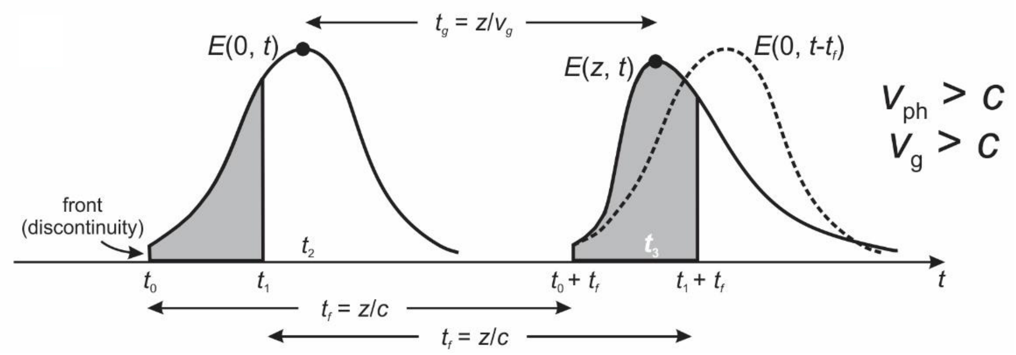

Supplementary Fig. 2. Superluminal pulse propagation in the outer shell of the tachyonic cloak (upper part of Fig. 1B). Shown are the input pulse at the time-instant t_2 where it enters the cloak, and the same pulse at a later time-instant, t_3, when it exits the cloak (cf. upper part of Fig. 1B and Ref. [22]). As explained herein in this section, only the shaded parts of the pulses are causally connected. Note that the shaded part of the input pulse does not contain the peak of that pulse (at $t = t_2$), whereas the shaded part of the exit pulse does contain the peak of that pulse (at $t = t_3$) – i.e., the two peaks, the time-distance between which determines the group velocity, are not causally connected; hence, the group velocity of the pulse can be superluminal without violation of relativistic causality.

The characteristics of the input (incident on the cloak) and output (exiting the cloak) pulses are shown in Supplementary Fig. 2 above. We shall now prove these shown characteristics in the following, specifically showing that:

- The sharp/sudden front discontinuity of the input pulse, associated with the pulse's *signal velocity*, propagates with a velocity c;
- Only the shaded parts of the two pulses, as shown in Supplementary Fig. 2, are causally connected;
- The shadowed part of the input pulse does not contain the peak of that pulse, whereas the shadowed part of the exit pulse does contain the peak of that (exit) pulse.

To prove the first point, let us assume that $E(z = 0, t)$ is the electric-field value of the pulse entering the tachyonic cloak (at an arbitrary point $z = 0$) – cf. Suppl. Fig. 2 above. The input pulse is "turned on" at a time instant $t = t_0$, as shown in Suppl. Fig. 2, i.e. it is:

$E(0, t) = 0$ for $t < t_0$. Then, the electric-field value, $E(z, t)$, of the pulse exiting the cloak will simply be given by:

$$E(z,t) = \frac{1}{2\pi}\int_{-\infty}^{\infty} \mathrm{e}^{\mathrm{j}(\omega t - kz)} \hat{E}(0,\omega)\mathrm{d}\omega = \frac{1}{2\pi}\int_{-\infty}^{\infty} \mathrm{e}^{\mathrm{j}(\omega t - \omega t_0 - kz)} \mathrm{e}^{\mathrm{j}\omega t_0} \hat{E}(0,\omega)\mathrm{d}\omega, \quad \text{(S11)}$$

where k is the wavevector of the pulse inside the cloak, and $\hat{E}(0,\omega)$ is the Fourier transform of $E(0, t)$:

$$\hat{E}(0,\omega) = \int_{-\infty}^{\infty} \mathrm{e}^{-\mathrm{j}\omega t} E(0,t)\mathrm{d}t. \quad \text{(S12)}$$

We shall, now, show that $E(z, t) = 0$ for $t < t_0 + t_f = t_0 + z/c$, as shown in Suppl. Fig. 2. To this end, we note that the permittivity model describing the inverted (gain) medium in the outer shell of the tachyonic cloak (in its most general form that model is given by: $\varepsilon(\omega) = \varepsilon_0 + \varepsilon_0 f \omega_p^2/(\omega_0^2 - \omega^2 + \mathrm{j}\omega\gamma)$, with $f < 0$ being the oscillator strength, and ω_p the "plasma" frequency) leads to the wavevector $k(\omega)$ having poles only in the upper complex ω-plane, i.e. being analytic in the lower half plane, as shown in Supplementary Fig. 3 below. Thus, from complex integration we have:

$$E(z,t) = \frac{1}{2\pi}\int_{-\infty}^{\infty} \mathrm{e}^{\mathrm{j}(\omega t - \omega t_0 - kz)} \mathrm{e}^{\mathrm{j}\omega t_0} \hat{E}(0,\omega)\mathrm{d}\omega = \lim_{R\to\infty} \frac{1}{2\pi}\int_{H_R^-} \mathrm{e}^{\mathrm{j}(\omega t - \omega t_0 - kz)} \mathrm{e}^{\mathrm{j}\omega t_0} \hat{E}(0,\omega)\mathrm{d}\omega. \quad \text{(S13)}$$

The crucial point, now, is that on the semicircle H_R^- it is $|\omega| = R \to \infty$, hence the wavevector k will equal its free-space value, $k_0 = \omega/c$, because $\lim_{\omega\to\infty} \varepsilon(\omega) = \varepsilon_0$. Equation (S13) may, therefore, be re-written as:

$$E(z,t) = \lim_{R\to\infty} \frac{1}{2\pi}\int_{H_R^-} \mathrm{e}^{\mathrm{j}\omega(t - t_0 - z/c)} \mathrm{e}^{\mathrm{j}\omega t_0} \hat{E}(0,\omega)\mathrm{d}\omega. \quad \text{(S14)}$$

Since, as mentioned above, we wish to check the value of $E(z, t)$ for $t < t_0 + z/c$, i.e. since $t - t_0 + z/c < 0$, and under the reasonable assumption that on H_R^- it is $\mathrm{e}^{\mathrm{j}\omega t_0} \hat{E}(0,\omega) \to 0$

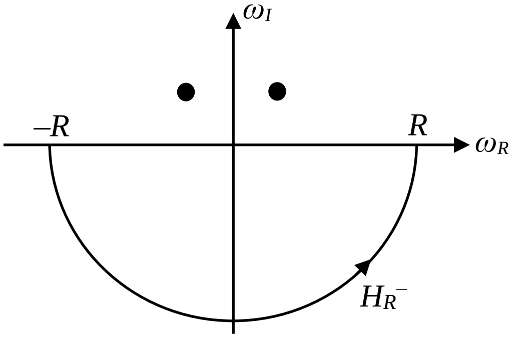

Supplementary Fig. 3. Complex ω-plane and associated contour of integration for the evaluation of the integral of Eq. (S13).

(for instance, for a causal signal $E(0,t) = e^{-\beta(t-t_0)} e^{j\omega(t-t_0)u(t-t_0)}$, with $\beta > 0$, it is $\mathrm{e}^{\mathrm{j}\omega t_0}\hat{E}(0,\omega) = 1/[\mathrm{j}(\omega - \omega_0 - \mathrm{j}\beta)]$, which indeed tends to zero for $|\omega| \to \infty$), it immediately follows from the Jordan lemma that the integral in Eq. (S14) vanishes, i.e. for $t < t_0 + t_f$ it is indeed $E(z, t) = 0$ as Suppl. Fig. 2 shows. Thus, the velocity with which the front discontinuity propagates (i.e., the *signal velocity*) is not larger than the speed of light in vacuum, c (i.e., relativistic causality is fully respected in the tachyonic cloak), since the signal-front delay is not larger than $t_f = z/c$.

Next, we assume that $q(z, t)$ is the impulse response of the cloak, given (as usual) by:

$$q(z,t) = \frac{1}{2\pi}\int_{-\infty}^{\infty} \mathrm{e}^{\mathrm{j}(\omega t - kz)}\mathrm{d}\omega\,. \tag{S15}$$

Based on similar arguments as before for the behaviour of the wavevector k on the complex ω-plane (cf. Suppl. Fig. 3), we may successively write:

$$q(z,t) = \lim_{R\to\infty}\frac{1}{2\pi}\int_{H_R^-} \mathrm{e}^{\mathrm{j}\omega(t-z/c)}\mathrm{d}\omega = .\lim_{R\to\infty}\frac{\sin(R(t-z/c))}{\pi(t-z/c)} = \delta(t-z/c)\,, \tag{S16}$$

from where we see that the tachyonic cloak satisfies the causality condition: $q(z, t) = 0$ for $t < t_f = z/c$.

We now note that, by the convolution theorem, the electric-field value of the exit pulse that has propagated through the cloak (and around the object) can, in addition to Eq. (S11), be written as:

$$E(z,t) = \int_{-\infty}^{\infty} q(z,t-t')E(0,t')\mathrm{d}t'\,. \tag{S17}$$

In this integral, it should be: $t - t' \geq z/c$ (causality condition for $q(z, t)$) and also: $t' \geq t_0$ (since $E(0, t')$ is “turned on” at $t = t_0$; cf. Suppl. Fig. 2), i.e. overall it should be: $t_0 \leq t' \leq t - z/c$, leading to the following expression for $E(z, t)$:

$$E(z,t) = \int_{t_0}^{t-z/c} q(z,t-t')E(0,t')\mathrm{d}t', \tag{S18}$$

with $t > t_0 + t_f = t_0 + z/c$.

Equation (S18), which has been obtained on the basis of the causality conditions dictating the propagation of a pulse in the tachyonic cloak, shows that the electric-field amplitude of the exiting pulse at times $t > t_0 + z/c$, i.e. the shaded part of the $E(z, t)$ pulse in Suppl. Fig. 2, is causally determined by the shaded part of the input $E(0, t)$ pulse. Thus, only the shaded parts of the two pulses shown in Suppl. Fig. 2 are causally connected, as we were intending to show. Furthermore, since in the tachyonic cloak it is $v_g > c$, the group delay $t_g = z/v_g$ will be smaller than the signal-front delay $t_f = z/c$ ($t_g < t_f$). Therefore, if the peak of the exiting pulse occurs at a time point $t = t_3$ (cf. Suppl. Fig. 2), it will be:

$$E(z,t_3) = \int_{t_0}^{t_3 - z/c} q(z,t_3 - t')E(0,t')\mathrm{d}t', \tag{S19}$$

i.e. $E(z, t_3)$ will be causally determined by values of the input $E(0, t)$ pulse in the time interval $[t_0, t_3 - z/c]$, which *does not* include the time-point $t = t_3 - t_g = t_3 - z/v_g$ (the time point when the peak of the input $E(0, t)$ pulse occurs) because $v_g > c$. Thus, the peak of the input $E(0, t)$ pulse is *not* causally connected to the peak of the exiting $E(z, t)$ pulse – as a result of which, the time interval between the two peaks (group delay t_g) can indeed be "superluminal" without violation of relativistic causality.

3. Recursive scattering transfer matrix formulation

An exact methodology for the scattering problem at hand can be obtained through a recursive T matrix analysis. Particularly, let us initially assume an incoming plane electromagnetic (EM) wave of frequency ω, expressed in a spherical-wave basis around the center of the scatterer as follows:

$$\mathbf{E_0}(\mathbf{r}) = \sum_{l,m} \left[\frac{i}{k} a^0_{Elm} \nabla \times j_l(kr)\mathbf{X}_{lm}(\hat{\mathbf{r}}) + a^0_{Hlm} j_l(kr)\mathbf{X}_{lm}(\hat{\mathbf{r}}) \right], \tag{S20}$$

where $k = \sqrt{\varepsilon\mu}\,\omega/c$ is the wavenumber, $l = 1,2,\dots$ and $m = -l, -l+1, \dots, l$ are the usual angular momentum indices, $\mathbf{X}_{lm}(\hat{\mathbf{r}})$ are the vector spherical harmonics, with $j_l(kr)$ being the spherical Bessel function and a^0_{Plm} are appropriate expansion coefficients with index $P = E, H$. A similar expansion can be written for the outgoing scattered wave by replacing the spherical Bessel function with outgoing Hankel functions:

$$\mathbf{E_{sc}}(\mathbf{r}) = \sum_{l,m} \left[\frac{i}{k} a^+_{Elm} \nabla \times h^+_l(kr)\mathbf{X}_{lm}(\hat{\mathbf{r}}) + a^+_{Hlm} h^+_l(kr)\mathbf{X}_{lm}(\hat{\mathbf{r}}) \right]. \tag{S21}$$

The spherical expansion coefficients of the scattered waves, denoted a^+_{Plm}, can be connected to the coefficients of an incoming incident wave a^0_{Plm} through the scattering T matrix: $a^+_{Plm} = T_{Plm;P'l'm'} a^0_{P'l'm'}$. For a spherical scatterer, the T matrix actually takes the form $T_{Plm;P'l'm'} = T_{Pl}\delta_{PP'}\delta_{ll'}\delta_{mm'}$, where:

$$T_{El} = \frac{j_l(k_1 r)\frac{\partial}{\partial r}[rj_l(kr)]\varepsilon_1 - j(kr)\frac{\partial}{\partial r}[rj_l(k_1 r)]\varepsilon}{h^+_l(kr)\frac{\partial}{\partial r}[rj_l(k_1 r)]\varepsilon - j(k_1 r)\frac{\partial}{\partial r}[rh^+_l(kr)]\varepsilon_1}\Big|_{r=S} \tag{S22}$$

$$T_{Hl} = \frac{j_l(k_1 r)\frac{\partial}{\partial r}[rj_l(kr)]\mu_1 - j(kr)\frac{\partial}{\partial r}[rj_l(k_1 r)]\mu}{h^+_l(kr)\frac{\partial}{\partial r}[rj_l(k_1 r)]\mu - j(k_1 r)\frac{\partial}{\partial r}[rh^+_l(kr)]\mu_1}\Big|_{r=S} \tag{S23}$$

We now, for our purposes, assume a multilayer spherical particle which consists of a spherical core and *N-1* concentric spherical shells. Particularly, let the radius of the central spherical core be S_1 and the outer radii of each additional concentric spherical shells be $S_n, n = 2,3, \ldots, N$. Clearly, $S_1 < S_2 < \cdots < S_N$. The permittivities and permeabilities of the successive media are assumed to be ε_n and μ_n, respectively, and it is $\varepsilon_{N+1} \equiv \varepsilon$, $\mu_{N+1} \equiv \mu$ for the host medium. The corresponding wavenumbers are $k_n = \sqrt{\varepsilon_n \mu_n}\omega/c$ and $k_{N+1} \equiv k = \sqrt{\varepsilon\mu}\omega/c$, where c is the velocity of light in vacuum. Again, fully analytical solutions are here feasible; however, it is preferable to use a recursive formulation owing to its simplicity. By requiring the continuity of the tangential components of the EM field at each spherical interface between the consecutive media, we can write $T^{(n)}$, i.e., the T matrix for a sphere that consists of the n first shells, embedded in the $(n+1)^{\text{th}}$ medium which, one assumes, extends to infinity. The $T^{(n)}$ matrix is written recursively in terms of $T^{(n-1)}$, which describes a sphere consisting of the $(n-1)$ first shells in the nth host medium, $T^{(n-1)}$. In that case, we may obtain:

$$T_{El}^{(n)} = \frac{A_E^{(n,n+1)} + T_{El}^{(n-1)} B_E^{(n,n+1)}}{C_E^{(n,n+1)} + T_{El}^{(n-1)} D_E^{(n,n+1)}} , \qquad \text{(S24)}$$

with

$$A_E^{(n,n+1)} = j_l(k_n r)\frac{\partial}{\partial r}[r j_l(k_{n+1} r)]\varepsilon_n - j_l(k_{n+1} r)\frac{\partial}{\partial r}[r j_l(k_n r)]\varepsilon_{n+1} \qquad \text{(S25)}$$

$$B_E^{(n,n+1)} = h_l^+(k_n r)\frac{\partial}{\partial r}[r j_l(k_{n+1} r)]\varepsilon_n - j_l(k_{n+1} r)\frac{\partial}{\partial r}[r h_l^+(k_n r)]\varepsilon_{n+1} \qquad \text{(S26)}$$

$$C_E^{(n,n+1)} = h_l^+(k_{n+1} r)\frac{\partial}{\partial r}[r j_l(k_n r)]\varepsilon_{n+1} - j_l(k_n r)\frac{\partial}{\partial r}[r h_l^+(k_{n+1} r)]\varepsilon_n \qquad \text{(S27)}$$

$$D_E^{(n,n+1)} = h_l^+(k_{n+1} r)\frac{\partial}{\partial r}[r h_l^+(k_n r)]\varepsilon_{n+1} - h_l^+(k_n r)\frac{\partial}{\partial r}[r h_l^+(k_{n+1} r)]\varepsilon_n . \qquad \text{(S28)}$$

A similar expression is also obtained for $T_{Hl}^{(n)}$, with the magnetic permeabilities μ_n, μ_{n+1} used in place of the dielectric functions, ε_n, ε_{n+1}, respectively. For non-spherical scatterers, the *T* matrix is not diagonal in the spherical-wave basis and the elements can be evaluated numerically by employing the extended-boundary-condition method [1], properly modified [2].

4. Exact solution for scattering by a two-layered sphere (cf. Fig. 2)

For a two-layered sphere, the total scattering cross section Q_t due to plane wave scattering can be computed exactly, without even requiring a recursive *T* matrix formulation, using analytic theory. Suppose that the spherical core has radius a, and its material is described by constitutive parameters $\varepsilon_1 = \varepsilon_{1r}\varepsilon_0$ and $\mu_1 = \mu_{1r}\mu_0$, where ε_0, μ_0 are the free-space permittivity and permeability, respectively, and ε_{1r}, μ_{1r} the respective relative values. The shell has inner radius a and outer radius b, and is described by

respective parameters ε_2 and μ_2. Assuming an $\exp(i\omega t)$ time convention, Q_t, in m^2, is then given by:

$$Q_t = \frac{2\lambda_0^2}{\pi}\sum_{n=1}^{\infty}\frac{n^2(n+1)^2}{2n+1}(|F_{1n}|^2 + |G_{1n}|^2), \tag{S29}$$

where λ_0 is the free-space wavelength. The general expressions for the expansion coefficients F_{mn} and G_{mn}, $m = \ldots, -2, -1, 0, 1, 2, \ldots$, $n = |m|, |m|+1, \ldots$, are here given by:

$$F_{mn} = \frac{j_n(k_0 b)T_{1n} + \frac{1}{Z_0 k_0 b}\frac{d[k_0 b j_n(k_0 b)]}{d(k_0 b)}T_{2n}}{-h_n(k_0 b)T_{1n} - \frac{1}{Z_0 k_0 b}\frac{d[k_0 b h_n(k_0 b)]}{d(k_0 b)}T_{2n}} a_{mn}, \tag{S30}$$

$$G_{mn} = \frac{\frac{1}{Z_0} j_n(k_0 b)S_{1n} + \frac{1}{k_0 b}\frac{d[k_0 b j_n(k_0 b)]}{d(k_0 b)}S_{2n}}{-\frac{1}{Z_0} h_n(k_0 b)S_{1n} - \frac{1}{k_0 b}\frac{d[k_0 b h_n(k_0 b)]}{d(k_0 b)}S_{2n}} b_{mn}, \tag{S31}$$

where:

$$\begin{aligned} T_{1n} = {} & j_n(k_1 a)\frac{1}{Z_2 k_2 b}\frac{d[k_2 b j_n(k_2 b)]}{d(k_2 b)}\frac{1}{Z_2 k_2 a}\frac{d[k_2 a y_n(k_2 a)]}{d(k_2 a)} + j_n(k_2 a)\frac{1}{Z_1 k_1 a}\frac{d[k_1 a j_n(k_1 a)]}{d(k_1 a)}\frac{1}{Z_2 k_2 b}\frac{d[k_2 b y_n(k_2 b)]}{d(k_2 b)} \\ & - j_n(k_1 a)\frac{1}{Z_2 k_2 a}\frac{d[k_2 a j_n(k_2 a)]}{d(k_2 a)}\frac{1}{Z_2 k_2 b}\frac{d[k_2 b y_n(k_2 b)]}{d(k_2 b)} - y_n(k_2 a)\frac{1}{Z_1 k_1 a}\frac{d[k_1 a j_n(k_1 a)]}{d(k_1 a)}\frac{1}{Z_2 k_2 b}\frac{d[k_2 b j_n(k_2 b)]}{d(k_2 b)}, \end{aligned} \tag{S32}$$

$$\begin{aligned} T_{2n} = {} & -j_n(k_1 a)j_n(k_2 b)\frac{1}{Z_2 k_2 a}\frac{d[k_2 a y_n(k_2 a)]}{d(k_2 a)} + j_n(k_2 b)y_n(k_2 a)\frac{1}{Z_1 k_1 a}\frac{d[k_1 a j_n(k_1 a)]}{d(k_1 a)} \\ & - j_n(k_2 a)y_n(k_2 b)\frac{1}{Z_1 k_1 a}\frac{d[k_1 a j_n(k_1 a)]}{d(k_1 a)} + j_n(k_1 a)y_n(k_2 b)\frac{1}{Z_2 k_2 a}\frac{d[k_2 a j_n(k_2 a)]}{d(k_2 a)}, \end{aligned} \tag{S33}$$

$$\begin{aligned} S_{1n} = {} & \frac{1}{Z_1} j_n(k_1 a)\frac{1}{k_2 b}\frac{d[k_2 b j_n(k_2 b)]}{d(k_2 b)}\frac{1}{k_2 a}\frac{d[k_2 a y_n(k_2 a)]}{d(k_2 a)} + \frac{1}{Z_2} j_n(k_2 a)\frac{1}{k_1 a}\frac{d[k_1 a j_n(k_1 a)]}{d(k_1 a)}\frac{1}{k_2 b}\frac{d[k_2 b y_n(k_2 b)]}{d(k_2 b)} \\ & - \frac{1}{Z_1} j_n(k_1 a)\frac{1}{k_2 a}\frac{d[k_2 a j_n(k_2 a)]}{d(k_2 a)}\frac{1}{k_2 b}\frac{d[k_2 b y_n(k_2 b)]}{d(k_2 b)} - \frac{1}{Z_2} y_n(k_2 a)\frac{1}{k_1 a}\frac{d[k_1 a j_n(k_1 a)]}{d(k_1 a)}\frac{1}{k_2 b}\frac{d[k_2 b j_n(k_2 b)]}{d(k_2 b)}, \end{aligned} \tag{S34}$$

$$S_{2n} = -\frac{1}{Z_1} j_n(k_1 a) \frac{1}{Z_2} j_n(k_2 b) \frac{1}{k_2 a} \frac{d[k_2 a y_n(k_2 a)]}{d(k_2 a)} + \frac{1}{Z_2} j_n(k_2 b) \frac{1}{Z_2} y_n(k_2 a) \frac{1}{k_1 a} \frac{d[k_1 a j_n(k_1 a)]}{d(k_1 a)}$$
$$-\frac{1}{Z_2} j_n(k_2 a) \frac{1}{Z_2} y_n(k_2 b) \frac{1}{k_1 a} \frac{d[k_1 a j_n(k_1 a)]}{d(k_1 a)} + \frac{1}{Z_1} j_n(k_1 a) \frac{1}{Z_2} y_n(k_2 b) \frac{1}{k_2 a} \frac{d[k_2 a j_n(k_2 a)]}{d(k_2 a)},$$

(S35)

and a_{mn}, b_{mn} are the expansion coefficients of the incident plane wave. In the above expressions, j_n is the spherical Bessel function, y_n is the spherical Neumann function, and h_n is the spherical Hankel function of the second kind (the superscript (2) is omitted for simplicity). Furthermore, $Z_j = \sqrt{\mu_j / \varepsilon_j}, j = 0,1,2$, is the medium impedance, $k_0 = 2\pi / \lambda_0$ is the free-space wavenumber, $k_1 a = a / b\sqrt{\varepsilon_{1r}\mu_{1r}} k_0 b$, $k_2 a = a / b\sqrt{\varepsilon_{2r}\mu_{2r}} k_0 b$, and $k_2 b = \sqrt{\varepsilon_{2r}\mu_{2r}} k_0 b$. In this formulation, the plane wave is expanded in complex spherical vector wave functions $\mathbf{M}_{mn}^{(1)}$ and $\mathbf{N}_{mn}^{(1)}$ in the form $\mathbf{E}^{inc}(\mathbf{r}) = \sum_{m=-\infty}^{\infty} \sum_{n=|m|}^{\infty} [a_{mn}\mathbf{M}_{mn}^{(1)}(k_0, \mathbf{r}) + b_{mn}\mathbf{N}_{mn}^{(1)}(k_0, \mathbf{r})]$ [3]. For an isotropic spherical scatterer the angle of incidence and the polarization do not play a particular role. Assuming $\mathbf{E}^{inc}$ is $\mathbf{e}_x$-polarized and impinging from the negative towards the positive z-axis, only the $m = \pm 1$ terms survive, and we thus have: $a_{-1,n} = (-i)^{n+1}(2n+1)/2$, $a_{1,n} = a_{-1,n} / [n(n+1)]$, $b_{-1,n} = -a_{-1,n}$, and $b_{1,n} = a_{1,n}$. The scattering by the single sphere (uncloaked case) can be obtained directly by the present formulation, simply by letting $a \to 0$.

5. Performance of the tachyonic cloak for different object sizes and shapes

Supplementary Figure 4 below presents the normalized radar cross section (RCS) of a tachyonic cloak for different radii of an uncloaked (red) and cloaked (blue) spherical object, starting with an initial radius r_0. In all cases the incident wavelength is 600 nm. We may note that, as expected from the discussion in the main body of the work (bandwidth $BW = v_g/(Q \cdot \Delta\ell)$), for the mildly (realistic) superluminal group velocities used in all cases herein ($v_g|^{\text{outer-shell}}$ ~2.236c), i.e., with the group velocity in the outer layer being approximately *twice* larger than the speed of light in vacuum, the ultrabroadband (across the visible regime) bandwidth performance of the cloak can be maintained for sizes of the object that can, accordingly, approximately *double* before discriminable scattering ensues (i.e., with $\Delta\ell$ doubling, the superluminal group velocity v_g in the outer layer should also be, approximately, double the speed of light in vacuum, so that *BW* can be maintained large).

Using this methodology, one may also cloak an arbitrary shaped object – in this case (Suppl. Fig. 5 below), a randomly oriented Si spiral. In particular, as shown in Suppl. Fig. 5(b), the method can be used for cloaking, over extremely broad bandwidths, *hollow* metallic/plasmonic 3D spheres, inside which one may place a completely arbitrary object. We note that this manner for cloaking arbitrarily shaped objects was first proposed in the

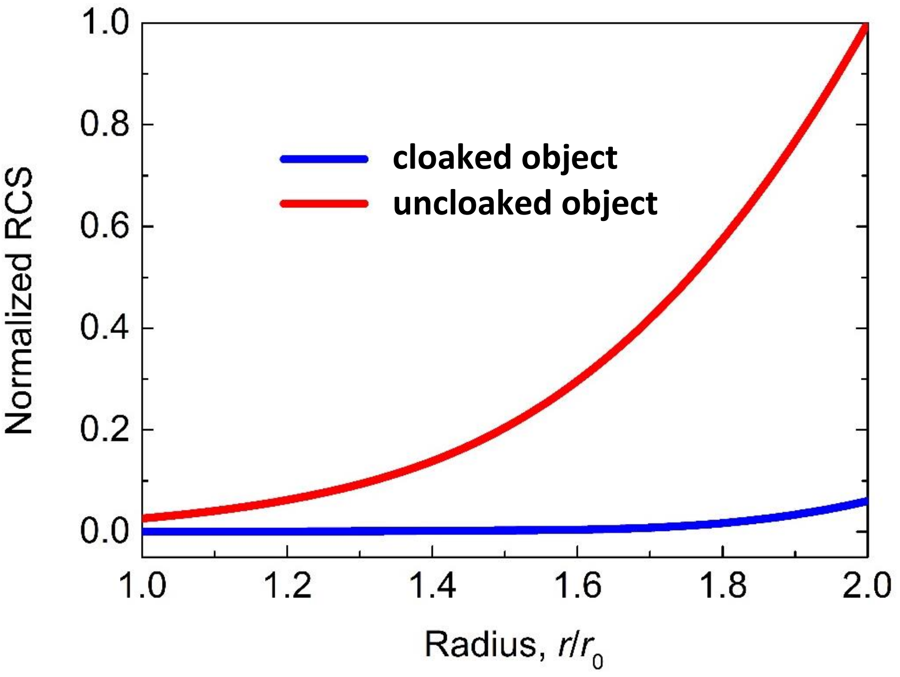

Supplementary Fig. 4. Performance of a tachyonic cloak (normalized ‘radar’ cross section, RCS) for different object sizes, for the realistic case where $v_g|^{\text{outer-shell}}$ ~2.236c.

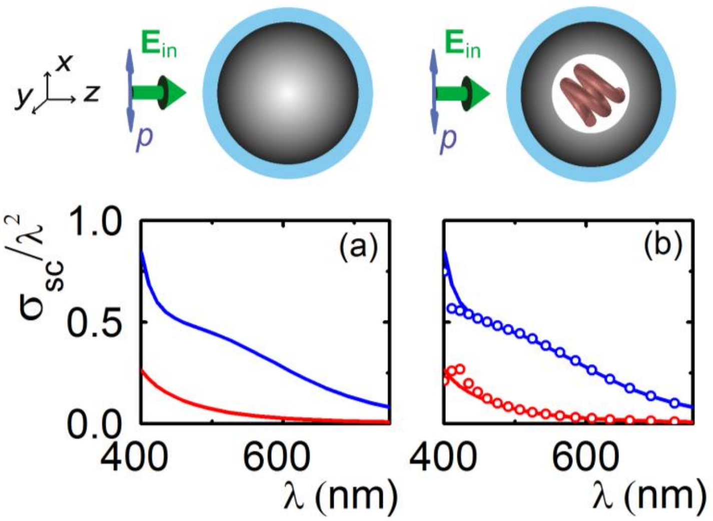

Supplementary Fig. 5. Tachyonic cloaking of an arbitrarily shaped object. (a) Normalized scattering cross section of an uncloaked (blue line) and cloaked (red line) plasmonic (Ag) spherical object shown in the top inset graphic. The thickness of the cloak is, here too, only 10 nm, and inside it the average (dispersive) group velocity is ten times largen than the speed of light in vacuum (<v_g> ~10c). (b) The solid lines show the normalized scattering cross section of an uncloaked (blue line) and cloaked (red line) *hollow* plasmonic (Ag) spherical shell, while the open symbols present the normalized scattering cross section of an uncloaked (blue symbols) and cloaked (red symbols) hollow plasmonic (Ag) spherical shell *with a Si spiral placed arbitrarily inside it*, as shown in the top inset.

original ‘transformation optics’ invisibility techniques [6, 7], where the innermost interior layer of the cloaks deployed therein was an impenetrable, perfect electric conductor (PEC), but those designs were fundamentally narrowband owing to (as explained in the main manuscript) the use of sub-luminal ($v_g < c$) light in the cloaks’ layers. In contrast, our present methodology allows for cloaking, in 3D, arbitrarily shaped objects across (almost) the entire visible band (cf. Suppl. Fig. 5(b)) by making use of realistic, broadband fast-light media, where $v_g > c$ over the required frequency bandwidth. Furthermore, the ‘see through’ functionality is, herein, still preserved since the thin (thickness smaller than or compared to the skin depth) hollow plasmonic object, at visible wavelengths, allows appreciable lightwave energy to still penetrate and pass through it.

Supplementary references

[1] M. I. Mishchenko, L. D. Travis, and A. A. Lacis, *Scattering, Absorption, and Emission of Light by Small Particles* (Cambridge University Press, Cambridge, 2002).

[2] N. Stefanou, C. Tserkezis, and G. Gantzounis, "Plasmonic excitations in ordered assemblies of metallic nanoshells," in *Photonic Crystal Materials and Devices VIII*, Vol. 6989, International Society for Optics and Photonics, 2008.

[3] Craig F. Bohren and Donald R. Huffman, *Absorption and scattering of light by small particles* (John Wiley & Sons, 2008).
